# Surfactant-controlled composition and crystal structure of manganese(II) sulfide nanocrystals prepared by solvothermal synthesis

**DOI:** 10.3762/bjnano.6.238

**Published:** 2015-12-07

**Authors:** Elena Capetti, Anna M Ferretti, Vladimiro Dal Santo, Alessandro Ponti

**Affiliations:** 1 Laboratorio di Nanotecnologie, Istituto di Scienze e Tecnologie Molecolari, Consiglio Nazionale delle Ricerche, via G. Fantoli 16/15, 20138 Milano, Italy,; 2 Istituto di Scienze e Tecnologie Molecolari, Consiglio Nazionale delle Ricerche, via C. Golgi 19, 20133 Milano, Italy

**Keywords:** manganese oxide, manganese sulfide, nanocrystal, polymorphism control, solvothermal synthesis, sulfur, surfactant

## Abstract

We investigated how the outcome of the solvothermal synthesis of manganese(II) sulfide (MnS) nanocrystals (NCs) is affected by the type and amount of long chain surfactant present in the reaction mixture. Prompted by a previous observation that a larger than stoichiometric amount of sulfur is required [Puglisi, A.; Mondini, S.; Cenedese, S.; Ferretti, A. M.; Santo, N.; Ponti A. *Chem. Mater. ***2010**, *22*, 2804–2813], we carried out a wide set of reactions using Mn(II) carboxylates and Mn_2_(CO)_10_ as precursors with varying amounts of sulfur and carboxylic acid. MnS NCs were obtained provided that the S/Mn ratio was larger than the L/Mn ratio, otherwise MnO NCs were produced. Since MnS can crystallize in three distinct phases (rock salt α-MnS, zincblende β-MnS, and wurtzite γ-MnS), we also investigated whether the surfactant affected the NC polymorphism. We found that MnS polymorphism can be controlled by appropriate selection of the surfactant. γ-MnS nanocrystals formed when a 1:2 mixture of long chain carboxylic acid and amine was used, irrespective of the presence of carboxylic acid as a free surfactant or ligand in the metal precursor. When we used a single surfactant (carboxylic acid, alcohol, thiol, amine), α-MnS nanocrystals were obtained. The peculiar role of the amine seems to be related to its basicity. The nanocrystals were characterized by TEM and electron diffraction; ATR-FTIR spectroscopy provided information about the surfactants adsorbed on the NCs.

## Introduction

Manganese(II) sulfide (MnS) is a wide bandgap (*E*_g_ ≈ 3 eV) [1], p-type, antiferromagnetic semiconductor that crystallizes in three distinct phases: cubic α-MnS (rock salt), cubic β-MnS (zincblende), and hexagonal γ-MnS (wurtzite). In α-MnS, sulfide anions form an fcc lattice and manganese cations fill all of the octahedral voids. β-MnS also has an fcc lattice of S^2−^ ions but the Mn^2+^ ions occupy half of the tetrahedral voids. In γ-MnS, the S^2–^ ions form an hcp lattice and the Mn^2+^ ions again occupy half of the tetrahedral voids. α-MnS is the thermodynamically stable form and is found in manganese ore beds as the mineral alabandite, which has been known since the early 19th century [2]. More recently, naturally occurring minerals with β- and γ-MnS structure have been described. Rambergite (γ-MnS) was discovered in 1996 in Sweden [3]. In 2012, extraterrestrial browneite (β-MnS) was found in a meteorite collected in Poland [4]. All three forms of MnS display antiferromagnetic order [5] with a Néel temperature of ≈80 K (γ-MnS), ≈100 K (β-MnS), and 154 K (α-MnS) [6]. The interesting physical properties and the rich polymorphism prompted research on MnS nanocrystals (NCs) in view of applications as photoluminescent components [7], photoreduction catalysts [8], anode materials in lithium-ion batteries [9], and supercapacitor materials [10].

Although MnS NCs have been synthesized by different methods, including chemical vapor deposition [11–13] and hydrothermal [14–16] methods, here we focus on NCs synthesized by solvothermal methods, which usually allow more experimental flexibility and improved NC control. Whereas reports on the solvothermal synthesis of β-MnS NCs are rare [17], several syntheses of α-MnS [18–27] and γ-MnS [19,22,26–29] NCs have been described.

Diverse reagents were used to introduce sulfur in the reaction mixture: sulfur-containing Mn complexes [19–21,27], organic (thiourea [16], thioacetamide [26], bistrimethylsilylsulfide [18], dodecanethiol [30]) and inorganic (sulfur [22–24,28], ammonium sulfide [29]) compounds. The S/Mn molar ratio varied greatly, ranging from (1/3):1 to 4:1, but in most cases S/Mn ≥ 1. Use of S/Mn < 1 was successful when carboxylic acids were not present in the reaction mixture. In a previous investigation of the solvothermal synthesis of MnS NCs from Mn(II) oleates [23], it was shown that in the absence of free surfactants, an excess of sulfur (S/Mn ≥ 2) is needed to avoid the formation of MnO along with (or in place of) MnS NCs. We further pursued this investigation aiming at finding the sulfur concentration required to produce MnS NCs when different Mn precursors are used and free surfactants are added.

In the majority of literature reports that the synthesis of MnS NCs leads to structurally pure products, that is, α-, β- or γ-MnS NCs. Of course, this is advantageous as different crystal phases display different physical properties. However, in contrast to size and shape control, relatively little attention has been paid to the factors controlling the crystal structure of MnS NCs. The reaction temperature is of course a key physical parameter since high temperature usually favors achievement of chemical equilibrium and the most stable phase. It was early described that heating manganese(II) diethyldithiocarbamate in hexadecylamine at low temperature (120–150 °C) gave multipods comprising γ-MnS arms stemming from a β-MnS core, whereas 30 nm α-MnS cubes were obtained at 180 °C [19]. A more detailed investigation gave similar results for the reaction of manganese(II) chloride and thioacetamide in oleylamine, confirming the key role of temperature [26]. These authors also described the β→α and γ→α phase transformation of MnS NCs subject to high pressure. The formation of α- vs γ-MnS NCs was also shown to depend on the heating rate (15 °C/min: α-MnS; 25 and 35 °C/min: γ-MnS) when manganese(II) diethyldithiocarbamate was heated in octadecene at 320 °C in the presence of a large excess of both oleic acid and oleylamine (100:1 with respect to Mn) [27].

Polymorphism control could also be achieved by chemical means. By reacting manganese(II) chloride and thiourea in an autoclave (*T* = 190 °C) for 12 h, 30 nm α-MnS NCs were prepared using water as a solvent, whereas when the solvent was benzene, γ-MnS rods (*d* = 40–100 nm, *l* = 250–700 nm) were obtained [16]. The reaction of manganese(II) nitrate with elemental S in octadecylamine at 200 °C gave 50 nm α-MnS hexagons at high S concentration, whereas γ-MnS rods (*d* ≈ 50 nm) resulted at low S concentration [22]. The hydrothermal reaction of manganese(II) chloride with sodium sulfide at 180 °C yielded 200 nm α-MnS octahedral NCs but γ-MnS rods (*d* = 200–300 nm, *l* = 1.0–1.5 μm) were obtained when a large excess of hydrazine was added [15]. In summary, the control of the crystal structure of MnS NCs was achieved by varying the solvent, the amount of sulfur, or adding a reducing agent such as hydrazine. The effect of surfactant type on the crystal phase of MnS NCs has not yet been studied, to the best of our knowledge.

In this paper, we report on the control of the composition and crystal structure of MnS NCs obtained by solvothermal decomposition of sulfur-free manganese precursors in the presence of elemental sulfur and long chain surfactants. In particular, we will focus on two issues: (1) the effect of surfactant (L) and sulfur (S) on the formation of either MnO or MnS NCs and (2) the effect of the surfactant on the polymorphism of MnS NCs. We will show that the formation of MnS vs MnO NCs depends, in a nontrivial manner, on both S/Mn and L/Mn molar ratios. Additionally, we will show that the formation of α-MnS vs γ-MnS NCs can be controlled by selecting the surfactant(s) present in the reaction mixture. We unexpectedly found that α-MnS NCs are obtained when a single surfactant is present, irrespective of its polar end group, whereas γ-MnS NCs form when a mixture of long chain amine and carboxylic acid is used.

## Results and Discussion

In this paper we focus on how the composition and crystal structure of NCs are affected by (1) the choice of surfactant and (2) the concentration of sulfur and surfactant. In order to study the effect of the reactant type and concentration on the composition and polymorphism of the resulting NCs, all reactions were carried out using a common synthetic procedure, that is, heating to 320 °C (heating rate = 10 °C/min) in an octadecene solution of Mn precursor, S, and (possibly) surfactant, and finally, ageing for 1 h before cooling and isolating the resulting NCs.

### MnO vs MnS: The role of sulfur and surfactant

As anticipated in the Introduction, it was observed that when MnOl(OH) or MnOl_2_ (Ol - oleate) was decomposed in octadecene in the absence of any surfactant, a stoichiometric amount of sulfur led to a mixture of MnO and α-MnS NCs (pure batches of α-MnS NCs could be achieved using a S/Mn molar ratio ≥2 whereas pure MnO NCs were obtained using S/Mn ≤ 0.6) [23]. To gain a deeper insight, we carried out several reactions where a manganese precursor was thermally decomposed in octadecene containing varying amounts of sulfur and free surfactant (L).

**Figure 1:**
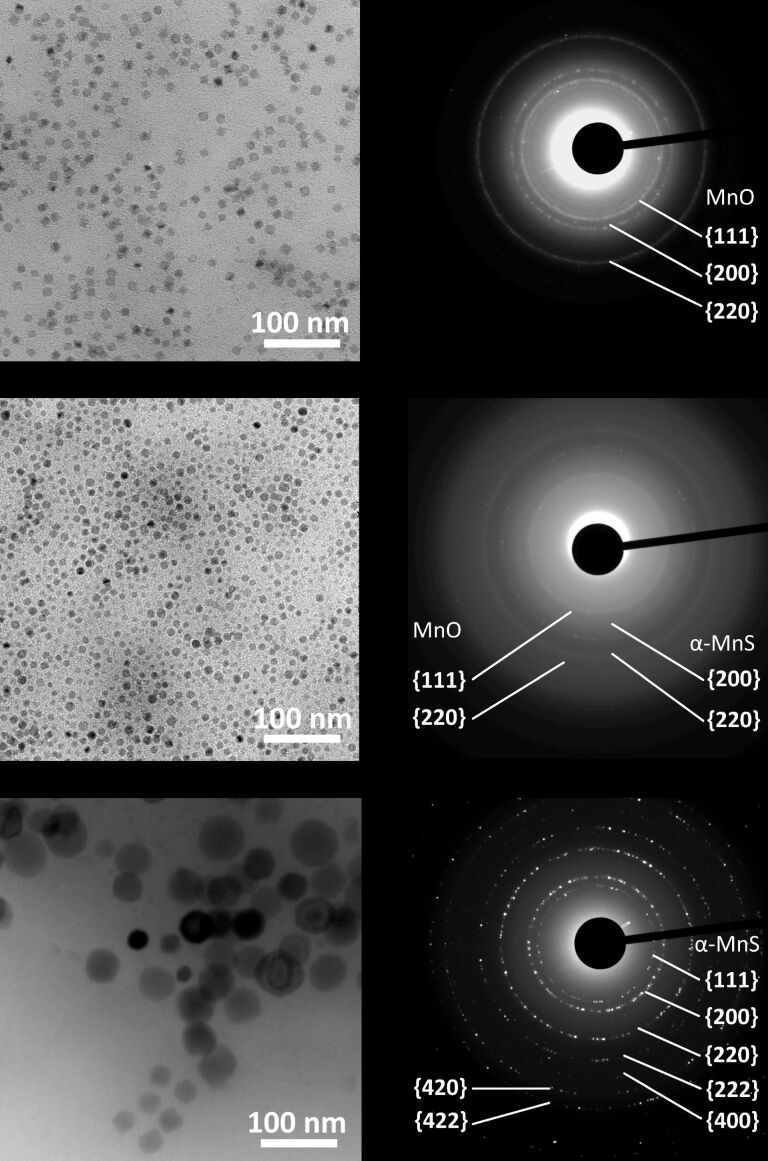
TEM images (left) and electron diffraction patterns (right) of NCs obtained by high temperature decomposition of Mn_2_(CO)_10_ with StAc/Mn = 2 and varying amounts of sulfur. (a) S/Mn = 1, MnO NCs; (b) S/Mn = 2, mixture of MnO and α-MnS NCs; (c) S/Mn = 4, α-MnS NCs.

**Figure 2:**
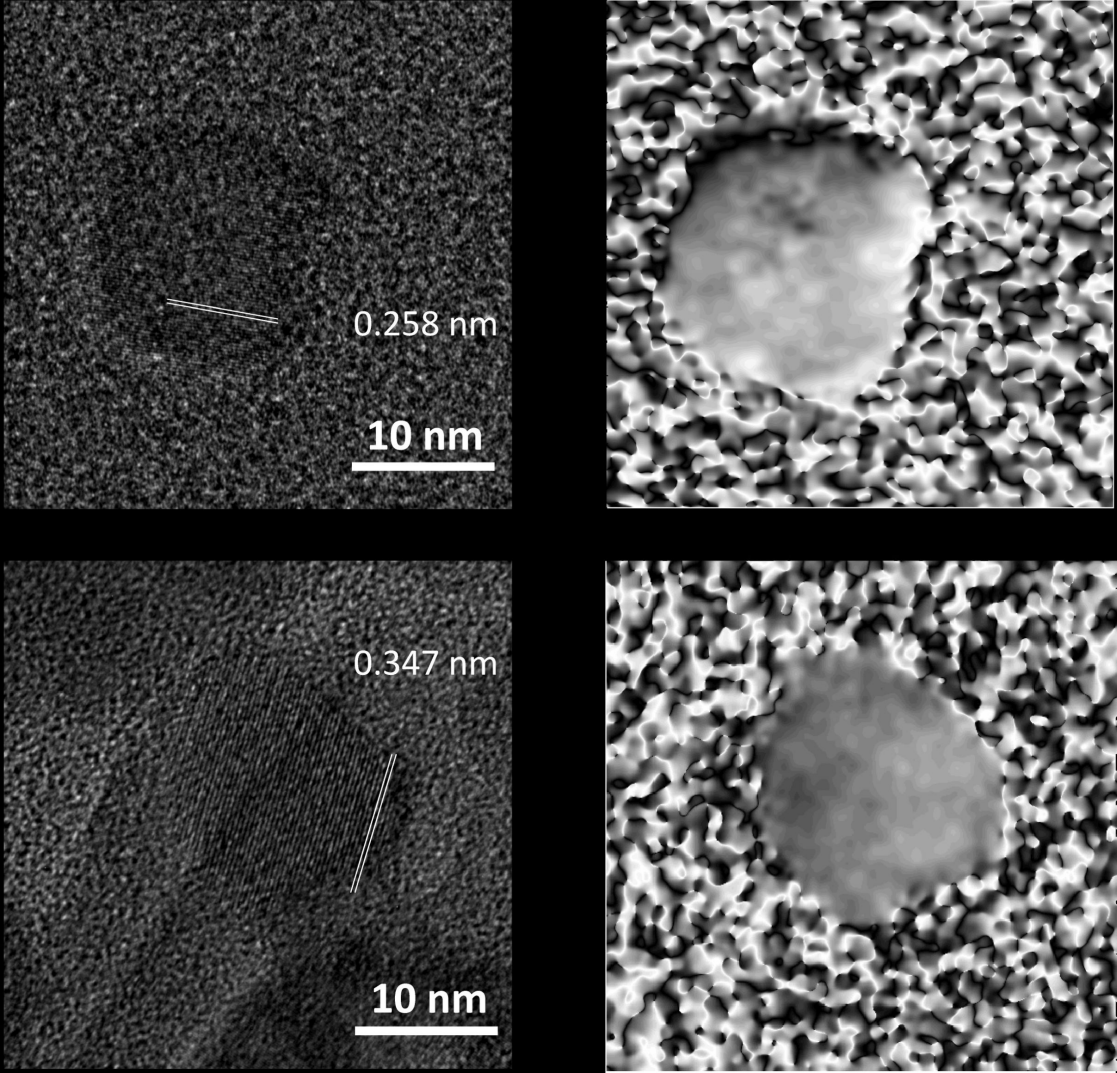
HRTEM images (left) and geometric phase analysis (GPA, right) of α-MnS and γ-MnS NCs. (a) α-MnS NCs prepared from Mn_2_(CO)_10_ in the presence of StAc (S/StAc/Mn = 2:4:1, where St = stearic acid) displaying 0.258 nm lattice fringes corresponding to {200} planes. (b) γ-MnS NC prepared from MnSt_2_ in the presence of HdAm (S/HdAm/Mn = 2:4:1, where HdAm = hexadecylamine) displaying 0.347 nm lattice fringes corresponding to {100} planes.

As manganese precursors we used manganese(II) carboxylates (Mn(OH)Ol, MnOl_2_, and MnSt_2_; abbreviations of chemical names can be found in the Experimental section) and an inorganic compound, Mn_2_(CO)_10_, which cannot release carboxylic acid upon decomposition. Transmission electron microscopy (TEM) images and electron diffraction (ED) patterns of representative NCs can be found in Figure 1. All ED patterns could be assigned to MnO or α-MnS NCs (or a mixture of both). The rock salt structure of MnS NCs was confirmed by HRTEM: Figure 2a displays lattice fringes separated by 0.258 nm that correspond to the {200} planes of the α-MnS structure. The geometric phase analysis (GPA) [31] showed that the NCs are single crystallites, almost free from lattice defects.

**Figure 3:**
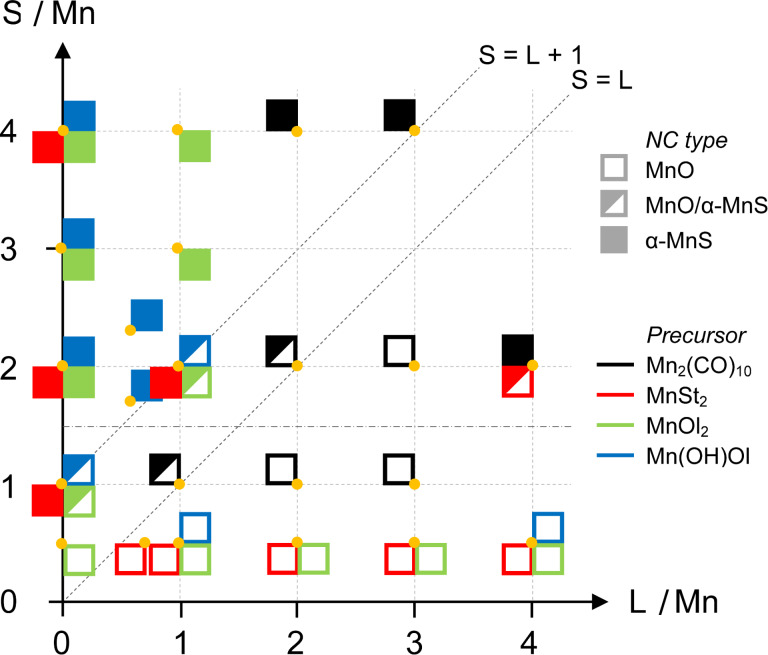
Pictorial representation of the NC outcome from the solvothermal synthesis involving the decomposition of different Mn precursors as a function of the L/Mn and S/Mn molar ratio. Each orange dot represents synthesis at the corresponding values of L/Mn and S/Mn. One to four squares arranged about each dot represent the resulting NCs when using different Mn precursors. The type of NC is encoded as follows: open squares: MnO NCs; half-filled squares: mixture of MnO and α-MnS NCs; solid squares: α-MnS NCs. The precursors are color coded as follows (counterclockwise from top left): black: Mn_2_(CO)_10_, red: MnSt_2_, green: MnOl_2_, blue: Mn(OH)Ol. The free surfactant, L, was StAc for Mn_2_(CO)_10_ and MnSt_2_, and OlAc for Mn(OH)Ol and MnOl_2_, respectively.

The chemical composition (MnO vs MnS) of the resulting NCs depended on the L/Mn and S/Mn molar ratios, as graphically summarized in Figure 3. Open (solid) squares indicate that MnO (MnS) NCs were obtained, and half-filled squares are used to represent a MnO/MnS NC mixture. The reactions carried out with no free surfactant correspond to data on the vertical axis of Figure 3. These results confirmed the previous conclusions about Mn(OH)Ol or MnOl_2_, in particular that a mixture of MnO and α-MnS NCs is obtained at a stoichiometric S/Mn = 1:1 ratio. The electron diffraction results gave no evidence that MnO*_x_*S_1−_*_x_* NCs formed despite that both MnO and α-MnS have a rock salt structure. We investigated a wide range of ageing times (30–180 min) and such a NC mixture was obtained in all cases, showing that interconversion of MnO and α-MnS NCs during ageing did not occur. Both of these findings are probably related to the largely different ionic radius of hexacoordinated O^2−^ (0.126 nm) and S^2−^ (0.170 nm) [32].

We also carried out reactions where a free surfactant (L) was added to the reaction mixture. In the case of Mn_2_(CO)_10_, we added free StAc. From Figure 3, one can see that we obtained a NC mixture when the molar amount of S and L were equal. Pure MnO NCs were prepared when the amount of S was lower than that of L. Conversely, when the amount of S was higher than L, the reaction yielded pure α-MnS NCs. In the case of Mn carboxylate precursors, the free surfactant was the corresponding carboxylic acid, i.e., OlAc for Mn(OH)Ol or MnOl_2_ and StAc for MnSt_2_. We again found that, in general, pure α-MnS NCs were obtained in sulfur-rich reactions and pure MnO NCs in surfactant-rich reactions. Reactions involving carboxylate precursors carried out using high S/Mn and L/Mn ratios often did not yield NCs.

On the whole, the resulting NC composition depends on sulfur and surfactant in a qualitatively simple way, which can be summarized as follows. When carboxylic acid is added as free surfactant, the amount of sulfur needed to produce MnS NCs increases. The plot in Figure 3 can be divided into two regions corresponding to MnO (bottom right) and α-MnS (top left) with a boundary running diagonally in an approximately linear way. Using Mn_2_(CO)_10_ as a precursor, it bisects the plot (S/L = 1:1). When Mn(OH)Ol or MnOl_2_ are used, the boundary is shifted towards the left side by about one unit (S/(L +1) = 1:1), suggesting that the oleate within the precursor plays a role in favoring the formation of MnO NCs. In this regard, MnSt_2_ is less efficient in that pure α-MnS NCs formed under conditions (L/Mn = 0:1, S/Mn = 1:1 and L/Mn = 1:1, S/Mn = 2:1) that led to α-MnS/MnO NC mixtures with the Mn mono- and dioleate precursors.

It was previously shown that at 180 °C elemental sulfur, which is present as cyclo-, catena- and polymeric species [33], reacts with 1-octadecene to produce more reactive H_2_S (yield ≈2/3), which reacts with cadmium oleate to give CdS NCs [34]. It is reasonable to think that H_2_S is the actual sulfur source in the present case, too. Whereas in the case of CdS it was established that the carboxylic acid did not react with H_2_S, in our case, the decreased sulfur availability at increased surfactant concentration suggests that reaction between RCOOH and H_2_S occurs. A possible reaction, which has long been known [35], is the transformation of RCOOH into the corresponding thio acid RC(O)SH by H_2_S. The occurrence of this (or a similar) reaction would explain the observed behavior (see Figure 3) provided that the product does not appreciably react with the Mn precursor. This explanation would also agree with the slightly different behavior of StAc and OlAc. Since MnSt_2_ decomposes at a higher temperature (310–360 °C) [36] than manganese(II) oleate (250–320 °C) [37], it can be argued that at the reaction temperature of 320 °C, MnSt_2_ decomposes and releases StAc more slowly and/or to a lesser extent than manganese(II) oleates so that more H_2_S is available for the formation of MnS NCs. One may wonder why the reaction of H_2_S with the carboxylic acid was not observed in the synthesis of CdS NCs. Our synthesis was carried out at a higher temperature (320 °C) and reagent concentration ([Mn] = 0.25 M, [S] and [L] = 0–1 M) than that of the CdS NCs (180 °C, [Cd] = 0.02 M, [S] = 0.01 M, [L] = 0.24 M), which may speed up the reaction of H_2_S with RCOOH. Additionally, the possible catalytic activity of Mn(II), a d^5^ ion with many possible oxidation states in comparison to d^10^ Cd(II), should not be overlooked.

A complete collection of the NC morphological data can be found in Supporting Information File 1. Here, it is sufficient to say that MnO NCs usually have an octahedral shape with size depending on the surfactant: it increases from Mn_2_(CO)_10_ (10–20 nm) to MnSt_2_ (up to 30 nm). α-MnS NCs are larger (10–65 nm), more polydisperse (15–35%) and have irregular (but in all cases convex) shape.

Recalling Figure 3, one can note that the reaction with S/Mn = 2:1 and L/Mn = 4:1 yielded α-MnS NCs or a mixture of α-MnS and MnO NCs when using Mn_2_(CO)_10_ or MnSt_2_, respectively. This outcome does not conform to the above view of a diagonal boundary separating α-MnS and MnO regions. We therefore decided to further investigate the S/L/Mn = 2:4:1 reaction in more detail by selecting L from a wide set of long chain surfactants of different chemical nature.

### The influence of free surfactant on the crystal structure of MnS NCs

**Figure 4:**
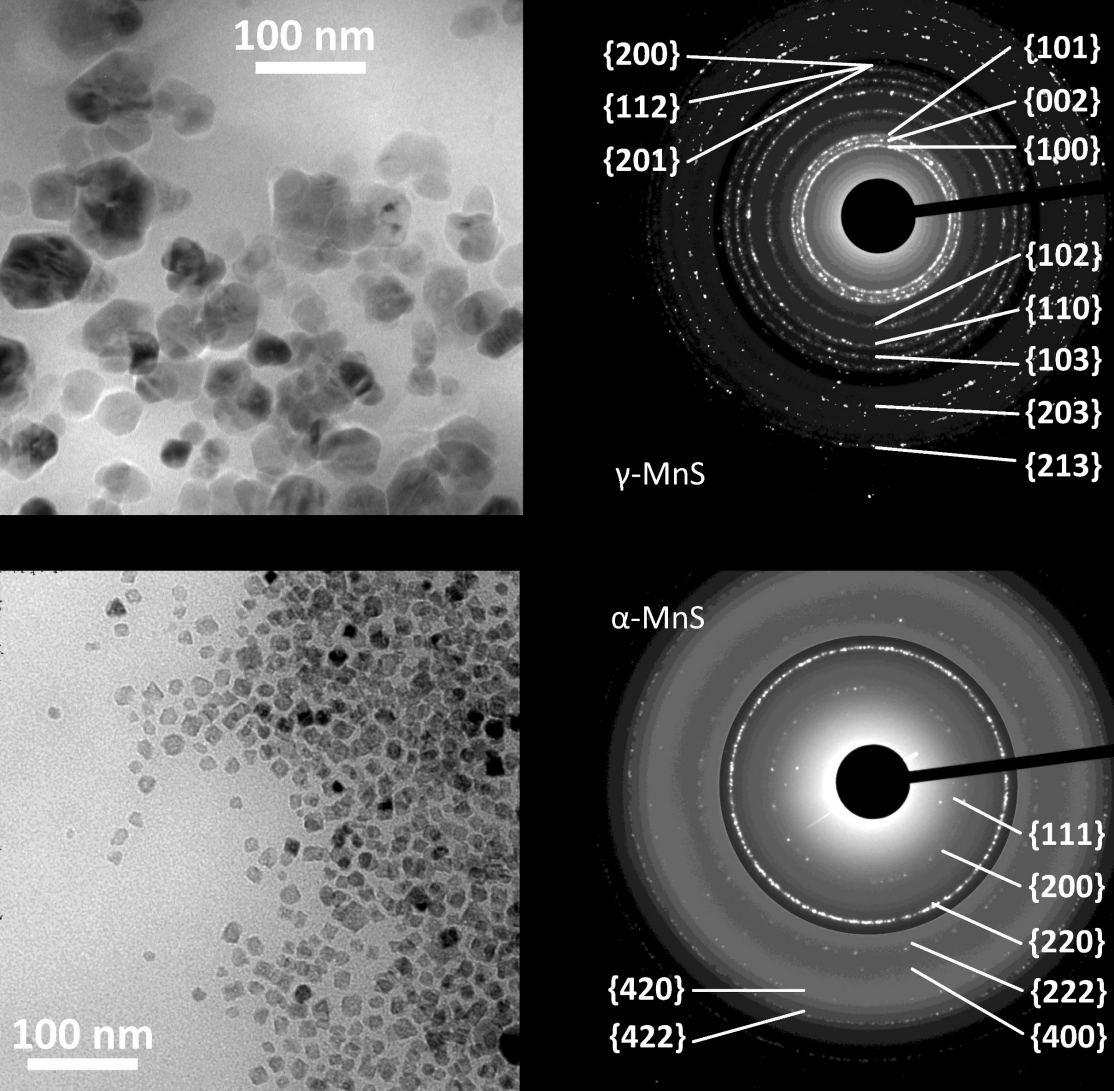
TEM images (left) and electron diffraction patterns (right) of NCs obtained by high temperature decomposition of MnSt_2_ with S/Mn = 2 and L/Mn = 4. (a) γ-MnS NCs synthesized using L = HdAm; (b) α-MnS NCs synthesized using L = DdTh (where DdTh = dodecanethiol). The contrast of the outer part of both diffraction patterns was digitally increased to show high-index rings.

In this subsection, we report on the synthetic outcome of the thermal decomposition of manganese(II) distearate (MnSt_2_) carried out with the S/L/Mn molar ratio fixed at 2:4:1. The long chain surfactant, L, was selected from a set including carboxylic acid, amine, alcohol, and thiol surfactants. This set allowed us to understand how basicity, nucleophilicity and ability to undergo condensation reaction with stearic acid affect the structure directing ability of the surfactant. The crystal structure of the resulting NCs was determined by analyzing the electron diffraction patterns. Selected ED patterns and TEM images are displayed in Figure 4. The identification of the crystal structure and the crystalline quality of the MnS NCs was confirmed by HRTEM images (Figure 2b). The crystal structure of the resulting NCs is summarized in Table 1 along with the morphological parameters.

**Table 1:** Properties of NCs synthesized by the thermal decomposition of MnSt_2_ with S/Mn = 2 and L/Mn = 4.

L	NC type	Shape^a^	Size (nm)^b^	Std. dev. (nm)

dodecylamine	γ-MnS	IRC	42 × 29	7 × 11
hexadecylamine	γ-MnS	IRC	55 × 38	15 × 10
octadecylamine	γ-MnS	IRC	55 × 38	12 × 9
oleylamine	γ-MnS	IRC	27 × 20	10 × 8
OlAm/DdTh^c^	γ-MnS	IRC	10–200	large
none^d^	α-MnS	spherical IRC	65 12 × 8	10 4 × 2
oleylalcohol	α-MnS	IRC	12 × 8	3 × 2
dodecanethiol	α-MnS	octahedral	12	2
stearic acid^d,e^	α-MnS MnO	spherical IRC	7 31 × 23	1 4 × 3

^a^IRC = irregular, rounded, convex shape; ^b^Mean size, in the case of anisotropic NCs, the mean size of the major and minor axis are reported; ^c^Oleylamine/dodecanethiol mixture (1:1 mol/mol); ^d^Two morphological classes observed in TEM images (See Supporting Information File 1); ^e^This sample also contains rare concave-shaped NCs (See Supporting Information File 1).

It is clear from Table 1 that amines direct the reaction toward γ-MnS NCs while all other free surfactants (or the absence thereof) lead to α-MnS NCs. This γ-directing effect, independent of the amine chain length and degree of saturation, seems to be related to the basicity of amines, which is highest within our surfactant set [38]. Nucleophilicity can be excluded since alkyl amines are less nucleophilic than sulfur compounds [39]. Similarly, the ability to undergo condensation reaction with carboxylic acids can be excluded since oleyl alcohol could react with stearic acid to give the corresponding ester. This was observed when long chain, ω-hydroxy carboxylic acids were employed in similar conditions [40]. Moreover, γ-MnS NCs were also obtained when the free surfactant was a 1:1 mol/mol mixture of OlAm and DdTh. This suggests that DdTh does not play a major role in the formation of MnS NCs, that is, it does not compete with H_2_S as a sulfur source, despite that it has often been used to synthesize metal sulfide NCs [41].

The NC morphology is summarized in Table 1, and TEM images can be found in Figure 4 and in Supporting Information File 1. γ-MnS NCs have similar morphology: they are irregular, rounded, and convex (IRC) NCs with an aspect ratio of 1.4–1.5; the size slightly depends on the particular amine present. The OlAm/DdTh surfactant mixture instead gave broadly polydisperse γ-MnS NCs. α-MnS NCs are in general smaller and have more regular shape than γ-MnS NCs. The octahedral shape of the α-MnS NCs obtained using L = DdTh is confirmed by the anomalous intensity of the electron diffraction rings (Figure 4b). Indeed, the outstanding {220} ring is signature of the highly crystalline texture induced by the octahedral NC shape [23].

### Role of stearate ligand in the precursor molecule

**Figure 5:**
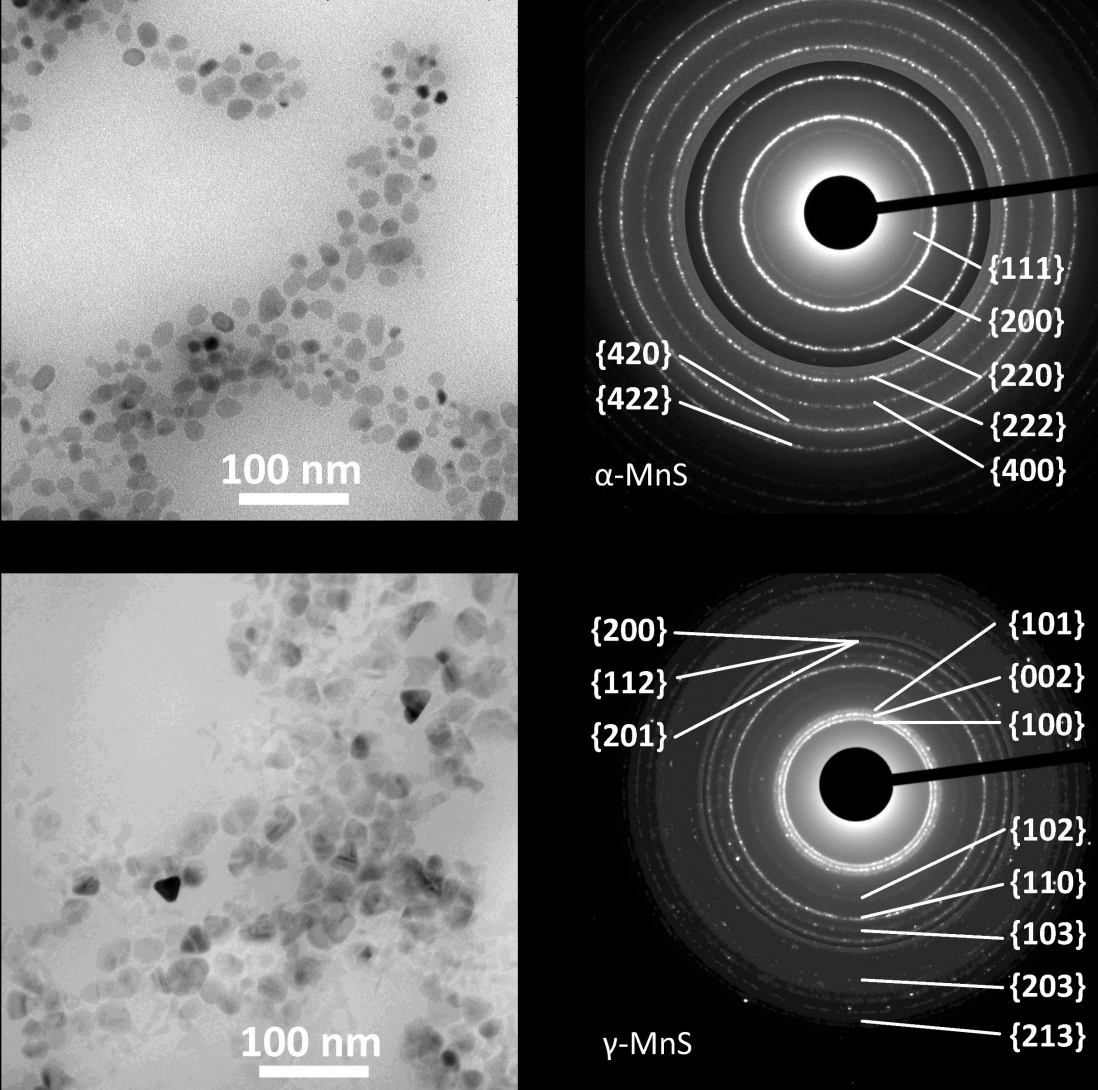
TEM images (left) and electron diffraction patterns (right) of NCs obtained by high temperature decomposition of Mn_2_(CO)_10_ with S/Mn = 2. (a) α-MnS NCs synthesized using HdAm (L/Mn = 4); (b) γ-MnS NCs synthesized using a mixture of StAc (L_acid_/Mn = 2:1) and HdAm (L_amine_/Mn = 4). The contrast of the outer part of both diffraction patterns was digitally increased to show the high index-rings.

To understand the role that the stearate ligand presents in the precursor in the above reactions leading to γ-MnS NCs, we carried out synthetic experiments similar to those described in the previous subsection, employing Mn_2_(CO)_10_ as a precursor and restricting ourselves to amines as a free surfactant. The main NC features are collected in Table 2, where TEM images and ED patterns of the L = HdAm case can be found in Figure 5a (see also the Supporting Information File 1). Unexpectedly, we found that α-MnS NCs were obtained in all cases, although a few γ-MnS NCs were intermixed with α-MnS NCs only when OlAm was used. The γ-directing effect of amines observed with MnSt_2_ vanished when Mn_2_(CO)_10_ was used, pointing at an important role of the stearate ligand in the polymorphism control. The NCs were smaller and had a lower aspect ratio than the corresponding ones prepared starting from MnSt_2_.

**Table 2:** Properties of NCs synthesized by the thermal decomposition of Mn_2_(CO)_10_ with S/Mn = 2 and L/Mn = 4.

L	NC type	Shape^a^	Size (nm)	Std. dev. (nm)

oleylamine	α-MnS (γ-MnS)	IRC	27 × 20	9 × 6
dodecylamine	α-MnS	IRC	18 × 14	6 × 4
hexadecylamine	α-MnS	IRC	22 × 16	4 × 3
octadecylamine	α-MnS	IRC	20 × 14	4 × 3

^a^IRC = irregular, rounded, convex shape.

To gain more insight into the role of stearate, we carried out reactions where Mn_2_(CO)_10_ was decomposed using a mixture of free surfactants including, as above, an amine (L_amine_/Mn = 4:1, L_amine_ = OlAm, DdAm, HdAm, OdAm) and a carboxylic acid in L_acid_/Mn = 2:1 molar ratio (L_acid_ = StAc, OlAc). The S/Mn ratio was 2, as before. These reactions mimicked those carried out using MnSt_2_ except that StAc is here present as a free surfactant and not as a ligand within the Mn precursor. The main features of the resulting NCs are collected in Table 3 and a TEM image and ED pattern of the L_amine_ = HdAm case can be found in Figure 5b (see also the Supporting Information File 1). We obtained γ-MnS NCs when saturated amines were used as a surfactant. This is evidence that both amine and stearic acid are required in the reaction mixture to direct the synthesis towards γ-MnS NCs. The irregularly shaped γ-MnS NCs were somewhat smaller than those prepared from MnSt_2_ and had a similar aspect ratio (1.35–1.45).

**Table 3:** Properties of NCs synthesized by the thermal decomposition of Mn_2_(CO)_10_ with S/Mn = 2 and using both amine and carboxylic acid as a free surfactant (L_amine_/Mn = 4:1, L_acid_/Mn = 2:1).

L_acid_	L_amine_	NC type	Shape^a^	Size (nm)	Std. dev. (nm)

stearic acid	oleylamine^b^	α-MnS	spherical IRC	33 15 × 11	6 4 × 3
stearic acid	dodecylamine	γ-MnS	IRC	24 × 18	9 × 7
stearic acid	hexadecylamine	γ-MnS	IRC	30 × 21	7 × 5
stearic acid	octadecylamine	γ-MnS	IRC	35 × 24	10 × 7
oleic acid	hexadecylamine^b^	γ-MnS	spherical spherical	11 28	2 7

^a^IRC = irregular, rounded, convex shape; ^b^Two morphological classes observed in TEM images (See Supporting Information File 1).

OlAm represented an exception in that α-MnS NCs were obtained. This could depend on the presence of a double bond in the OlAm chain, which is the main structural difference between OlAm and the other amines. To investigate this issue, we performed a similar reaction using a mixture of OlAc and HdAm, which yielded γ-MnS NCs. Thus, the presence of a double bond in the free surfactant chain does not favor the formation of α- vs γ-MnS NCs. Considering that OlAm also showed a peculiar behavior in the reaction where StAc was absent (see Table 2), one might be tempted to ascribe these differences to the low purity of commercially available OlAm [42].

The observation that both OlAc/HdAm and StAc/(saturated amines) surfactant mixtures direct the synthetic outcome towards γ-MnS NCs supports the view that the formation of γ-MnS NCs is insensitive to the structural details of both surfactants.

In conclusion, the data presented herein are evidence that to prepare γ-MnS NCs from a solution of elemental sulfur and a manganese precursor in octadecene, it necessary that the reaction mixture comprises both amine and carboxylic acid surfactants, otherwise α-MnS NCs are formed. Whether the carboxylic acid is present as free surfactant or as carboxylate ligand within the Mn precursor is irrelevant to the crystal structure of the resulting NCs.

### ATR-IR spectroscopy of MnS NCs

**Figure 6:**
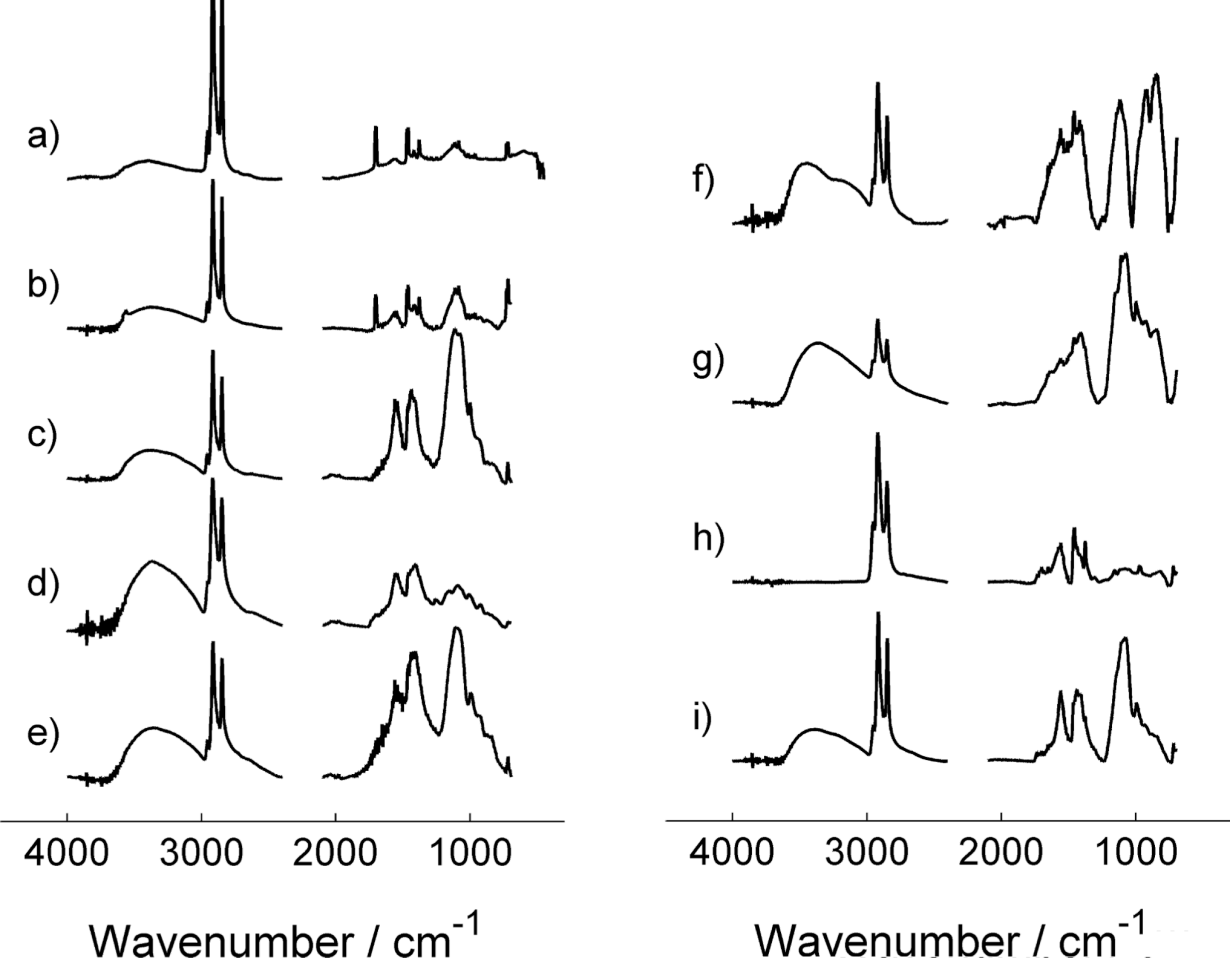
FTIR-ATR spectra of MnS NCs (selection of significant ranges). Left: spectra of NCs prepared using MnSt_2_ as a precursor with S/Mn = 2 and L/Mn = 4; L = StAc (a), DdTh (b), HdAm (c), OlAm (d), OlAm/DdTh 1:1 mixture (e). Right: spectra of NCs prepared using Mn_2_(CO)_10_ as a precursor with S/Mn = 2. Spectra (f) and (g) correspond to NCs prepared with amine surfactant only (L/Mn = 4:1); L = HdAm (f), OlAm (g). Spectra (h) and (i) correspond to NCs prepared using a mixture of StAc (L_acid_/Mn = 2:1) and amine surfactant (L_amine_/Mn = 4:1); L_amine_ = HdAm (h), OlAm (i).

The attenuated total reflectance infrared (ATR-IR) spectra of the NCs was recorded for several reactions (Tables 1–3) to investigate the organic species present on the NC surface at the end of the reaction. In particular, we analyzed NCs from reactions using both MnSt_2_ (L = HdAm, OlAm, DdTh, OlAm/DdTh, and StAc) and Mn_2_(CO)_10_ (L = HdAm, OlAm, StAc/HdAm, and StAc/OlAm) as a precursor (see Figure 6). For the sake of clarity, the ATR-IR results will be discussed in the same order as in previous subsections.

Using MnSt_2_ as a precursor, both StAc and DdTh gave α-MnS NCs. The spectra of these NCs are very similar (Figure 6a,b). The presence of both stearate and StAc is clearly discernible: two doublets ν_as_(C=O) and ν_s_(C=O) of carboxylate moiety are located at 1564, 1552 and 1471, 1462 cm^−1^, respectively, and a doublet of ν(C=O) of the COOH group is located at 1705 and 1699 cm^−1^. The bands located at 1462 and 1564 cm^−1^ closely resemble those of M^2+^ stearates. The occurrence of a splitting of ν(C=O) bands of StAc and stearates is reminiscent of the Davydov splitting occurring in highly ordered structures such as Langmuir–Blodgett films [43]. This suggests the formation of ordered layers on the NCs surface. Notably, the presence of strongly adsorbed StAc on metal sulfides surfaces, which could not be eliminated even with several washing cycles, is in agreement with previous papers: Hironaka et al. [44] reported on StAc adsorbing more strongly on sulfides, such as FeS, rather than on oxides. The presence of DdTh on NCs prepared by decomposing MnSt_2_ with free DdTh can be ruled out by the absence of absorption bands ascribable to DdTh and by the close similarity of this spectrum to the spectrum of NCs prepared in the presence of free StAc.

The NCs synthesized by decomposing MnSt_2_ in the presence of an amine (yielding γ-MnS NCs) gave similar spectra, where bands corresponding to both stearate and amine can be discerned. For example, when OlAm or HdAm were used (Figure 6c,d), the presence of the corresponding amines can be inferred from the band located at 720 cm^−1^ (ascribable to the γ_w_(NH_2_) wagging vibration [45]), while stearate originates two broad bands at 1551 and 1433 cm^−1^ (due to ν_s_(C=O) and ν_as_(C=O) of the carboxylate group). Hence, the NCs seem to be coated with both surfactants. The presence of oleylammonium or hexadecyammonium stearate salts cannot be excluded; however, there is no positive evidence, since the presence of carboxylate is not sufficient proof. Similar conclusions also apply to the spectrum of γ-MnS NCs prepared using a DdTh/OlAm mixture (Figure 6e).

We now turn to NCs prepared using Mn_2_(CO)_10_ as a precursor. We first note that the strong CO stretching absorption bands of the precursor (1976, 2010, 2045 cm^–1^) [46] were not detectable, indicating its absence in the product. When the latter was decomposed in the presence of either HdAm or OlAm alone, α-MnS NCs were unexpectedly obtained. These two samples (Figure 6f,g) show intense bands at 2955, 2920 and 2850 cm^−1^, originated by ν_s,as_(C–H) vibrations of aliphatic CH_2_ and CH_3_ groups, and two complex system of bands located between 800–1200 and 1300–1700 cm^−1^, respectively. The latter system, even if significantly broadened, closely resembles the 1600–1300 cm^–1^ spectral region of pure OlAm and HdAm (1467 cm^–1^: C–H bending, 1379 cm^–1^: C–C deformation). Conversely, the 800–1200 region is not easily attributable to any vibration of pure amines. We can conclude that these NCs are coated with the corresponding amine, as expected.

Finally, the spectra of the NCs obtained from Mn_2_(CO)_10_ using the mixture StAc/HdAm and StAc/OlAm (Figure 6h,i) closely resemble those observed for γ-MnS NCs prepared using MnSt_2_ with HdAm or OlAm, respectively. This is particularly true when the 1300–1600 cm^−1^ spectral region is considered. The coating of the NCs prepared using the surfactant mixture thus comprises stearate and amines, and the presence of ammonium salts could not be excluded nor confirmed. It also is noteworthy that there is no clear difference between the final coating of α-MnS and γ-MnS NCs. These spectra further confirm that it is immaterial that StAc is present as free surfactant or bound in the Mn precursor.

The C–H stretching of vinyl protons was not observed in NC spectra, including those prepared in the presence of unsaturated compounds only (e.g., Figure 6f). This behavior has been already observed, mainly when OlAc is involved, and is probably due the disappearance of the C=C double bond. Environmental effects leading to shifting/broadening of the vinyl C–H stretching band can be ruled out since the latter can be observed in close-packed OlAc monolayers [47].

In summary, NCs prepared in the absence of amines show the presence of both stearic acid and stearate. Conversely, when stearic acid and an amine were present in the reaction mixture, both stearates and amines could be detected in the NC coating. This is noteworthy since carboxylic acids are usually able to displace amines from the surface of inorganic NCs, where they bind as carboxylate anion. Unfortunately, a clear relationship between adsorbed species and the polymorphism control is not readily evident.

## Conclusion

In this work we have shown how the outcome of the solvothermal synthesis of MnO and MnS NCs can be controlled by varying and concentration of sulfur and surfactant and the nature of the latter. The chemical composition of the NCs (MnO vs MnS) can be predicted on the basis of the S/Mn and L/Mn molar ratios: MnS is obtained when S/Mn is larger than L/Mn. More interestingly, the polymorphism of the MnS NCs can be controlled by appropriate choice of the surfactant. α-MnS NCs are obtained when no or a single surfactant is present, irrespective of the chemical nature of the latter. When a 1:2 mixture of carboxylic acid and amine is used, γ-MnS NCs are instead obtained.

The α- vs γ-MnS control does not depend on the details of the chemical structure of amine and carboxylic acid and is also independent of whether the carboxylic acid is present as a free surfactant or is contained in the manganese precursor. The peculiar role of the amine (no other surfactant in combination with carboxylic acid is able to direct the synthesis to γ-MnS NCs) seems to be related to its basicity. The ATR-IR results provided some information about the surfactants stabilizing the final NCs: those prepared in absence of amines show the presence of both stearic acid and stearate, whereas in presence of stearic acid and an amine, both stearate and amine (or alkylammonium cation) could be detected although carboxylic acids are usually stronger ligands than amines.

The chemistry occurring in the reported reactions is quite complex, especially in the formation of γ-MnS NCs, where both stearic acid and amine are present. As we have seen, 1-octadecene can react with sulfur at 180 °C to produce H_2_S but it was also shown that long chain alkylamines react with sulfur following several two-step pathways, all of which produce H_2_S at 130 °C [48]. To add to the complexity, carboxylic acids may react with both alkylamines and H_2_S. It is clearly difficult to understand the peculiar chemistry leading to γ-MnS NCs as many reacting species are simultaneously present. However, two brief considerations can be made about the specificity of the amine/carboxylic acid mixture. First, we found that the carboxylic acid is equally effective both as a carboxylate ligand in the precursor and free acid. This could be explained by the formation of a manganese stearate intermediate when Mn_2_(CO)_10_ is heated in the presence of StAc, similar to the case of Fe(CO)_5_ and oleic acid that give an iron oleate intermediate in the synthesis of iron oxide NCs [49]. Second, the formation of γ-MnS NCs seems to be related to the basicity of amines but it only occurs when a carboxylic acid is also present. This suggests that monoalkylammonium carboxylate salts (R^1^–NH_3_^+^ R^2^–COO^−^), which were reported to form in an apolar solvent such as cyclohexane [45], may play a role in the polymorphism control. Unfortunately, ATR-IR measurements could not confirm (nor exclude) the presence of long chain ammonium stearates on the final NCs since their spectrum is very close to that of a mixture of stearate and amine molecules chemisorbed on the MnS surface.

## Experimental

### Materials

Manganese(II) chloride (reagent grade, ≥99%), tetramethylammonium hydroxide pentahydrate, sulfur powder (99.98%), dimanganese decacarbonyl (98%), oleic acid (90%), stearic acid (99%), oleylamine (technical grade, 70%), dodecylamine (98%), hexadecylamine (90%), octadecylamine (≥99%), dodecanthiol (98%), oleylalcohol (≥85%), tryoctylphosphine (97%), and octadecene (purum, ≥95.0%) were purchased from Sigma-Aldrich. Stearic acid (99%) was purchased from Calbiochem. All chemicals were used as received without further purification.

### Synthesis of manganese(II) distearate

Manganese distearate (MnSt_2_) was synthesized by a modification of the procedure reported in [23]. In a three-necked flask, a clear solution of of tetramethylammonium hydroxide pentahydrate (4.55 g, 50 mmol) and stearic acid (14.2 g, 50 mmol) in methanol (175 mL) was prepared and stirred under argon for 1 h at room temperature. Then, the mixture was cooled to 0 °C using an ice bath and a solution of anhydrous MnCl_2_ (3.15 g, 25 mmol) in methanol (100 mL) was slowly added. A white precipitate immediately formed, which was collected by filtration using a Buchner funnel previously cooled to −20 °C and washed with cold methanol (3 times, 10 mL). Drying under vacuum (*p* ≈ 10^−2^ torr) for 6 h gave MnSt_2_ as a white powder. The average yield was ≈50%. IR (KBr), ν (cm^−1^): 2955, 2917, 2849, 1552, 1468, 1440, 1320, 1113, 949, 721.

### Synthesis of manganese(II) monooleate

Manganese monooleate hydroxide (Mn(Ol)(OH)) was synthesized following the procedure reported in [23].

### Synthesis of MnX (X = S, O) NCs

The procedure to prepare MnX NCs is described with reference to the S/L/Mn = 2:4:1 case. In a two-necked flask, a solution of manganese(II) distearate (0.26 mmol, 0.25 M), sulfur (0.52 mmol, 0.50 M), and stearic acid (1.04 mmol, 1.00 M) in 1-octadecene was prepared. The solution was heated to 320 °C (heating rate = 10 °C/min) under nitrogen and magnetic stirring. After 1 h, the reaction mixture was cooled to room temperature. The NCs were precipitated by adding the reaction crude with a five-fold excess of ethanol and then collected by centrifugation (6000 rpm, 10 min). Next, the NCs were washed with ethanol and collected by centrifugation (three times) and then washed with acetone and collected by centrifugation (three times). The precipitate was finally dispersed in hexane (5–10 mL) by sonication (ultrasonic bath, 1 h, RT). The obtained NC dispersions are stable for several months.

Several variants of this procedure were carried out by changing:

1. the manganese precursor (manganese distearate (MnSt_2_) or Mn_2_(CO)_10_);
2. the S/Mn molar ratio (from 0:1 to 4:1);
3. the free surfactant (L = stearic acid (StAc), hexadecylamine (HdAm), dodecylamine (DdAm), octadecylamine (OdAm), oleylamine (OlAm), oleylalcohol (OlAl), or dodecanethiol (DdTh)); and
4. the L/Mn molar ratio (from 0:1 to 4:1).

### Characterization

Conventional transmission electron microscopy (TEM) images (medium resolution), high resolution TEM (HRTEM) images and electron diffraction (ED) patterns were recorded by a Zeiss LIBRA 200FE-HR TEM. The samples for microscopy were prepared by evaporating a drop of the nanocrystal dispersion in hexane on a carbon-coated TEM grid. ATR-FTIR spectra (4 cm^−1^ resolution, 50 scans) were collected using an ATR platform (Golden Gate, Specac) mounted in a spectrophotometer (FTS-40, Biorad) equipped with a KBr beam splitter and a MCT detector operating between 400 and 4000 cm^−1^. For ATR-FTIR spectroscopy, the purified NCs were further washed (2×) with a large excess of acetone and then collected by centrifugation (10000 rpm, 10 min). NCs with L = hexadecylamine were washed with CHCl_3_ to effectively remove the residual surfactant.

## Supporting Information

File 1 Additional TEM images and ED patterns of MnO and MnS nanocrystals.

## References

[1] Lokhande C D, Ennaoui A, Patil P S, Giersig M, Diesner K, Tributsch H (1998). Thin Solid Films.

[2] Papp G (2004). History of minerals, rocks and fossil resins discovered in the Carpathian region.

[3] Eriksson L, Kalinowski M P (2001). Acta Crystallogr, Sect E: Struct Rep Online.

[4] Ma C, Beckett J R, Rossman G R (2012). Am Mineral.

[5] Corliss L, Elliott N, Hastings J (1956). Phys Rev.

[6] Danielian A, Stevens K W H (1961). Proc Phys Soc, London.

[7] Viswanath R, Naik H S B, Kumar G S Y, Kumar P N P, Harish K N, Prabhakara M C (2014). Spectrochim Acta, Part A.

[8] Zhang X V, Martin S T, Friend C M, Schoonen M A A, Holland H D (2004). J Am Chem Soc.

[9] Beltran-Huarac J, Resto O, Carpena-Nuñez J, Jadwisienczak W M, Fonseca L F, Weiner B R, Morell G (2014). ACS Appl Mater Interfaces.

[10] Tang Y, Chen T, Yu S (2015). Chem Commun.

[11] Ge J, Li Y (2003). Chem Commun.

[12] Ge J-P, Wang J, Zhang H-X, Li Y-D (2004). Chem – Eur J.

[13] Kim D S, Lee J Y, Na C W, Yoon S W, Kim S Y, Park J, Jo Y, Jung M-H (2006). J Phys Chem B.

[14] An C, Tang K, Liu X, Li F, Zhou G, Qian Y (2003). J Cryst Growth.

[15] Gui Y, Qian L, Qian X (2011). Mater Chem Phys.

[16] Mu J, Gu Z, Wang L, Zhang Z, Sun H, Kang S-Z (2008). J Nanopart Res.

[17] Moloto N, Moloto M J, Kalenga M, Govindraju S, Airo M (2013). Opt Mater.

[18] Kan S, Felner I, Banin U (2001). Isr J Chem.

[19] Jun Y-w, Jung Y-y, Cheon J (2002). J Am Chem Soc.

[20] Pradhan N, Katz B, Efrima S (2003). J Phys Chem B.

[21] Tian L, Yep L Y, Ong T T, Yi J, Ding J, Vittal J J (2009). Cryst Growth Des.

[22] Wang D-S, Zheng W, Hao C-H, Peng Q, Li Y-D (2009). Chem – Eur J.

[23] Puglisi A, Mondini S, Cenedese S, Ferretti A M, Santo N, Ponti A (2010). Chem Mater.

[24] Tian Q, Tang M, Jiang F, Liu Y, Wu J, Zou R, Sun Y, Chen Z, Li R, Hu J (2011). Chem Commun.

[25] Yang X, Wang Y, Sui Y, Huang X, Cui T, Wang C, Liu B, Zou G, Zou B (2012). Langmuir.

[26] Yang X, Wang Y, Wang K, Sui Y, Zhang M, Li B, Ma Y, Liu B, Zou G, Zou B (2012). J Phys Chem C.

[27] Peng L, Shen S, Zhang Y, Xu H, Wang Q (2012). J Colloid Interface Sci.

[28] Joo J, Na H B, Yu T, Yu J H, Kim Y W, Wu F, Zhang J Z, Hyeon T (2003). J Am Chem Soc.

[29] Zhang H, Hyun B-R, Wise F W, Robinson R D (2012). Nano Lett.

[30] Choi S-H, An K, Kim E-G, Yu J H, Kim J H, Hyeon T (2009). Adv Funct Mater.

[31] Hÿtch M J, Snoeck E, Kilaas R (1998). Ultramicroscopy.

[32] Shannon R D (1976). Acta Crystallogr, Sect A: Found Adv.

[33] Meyer B (1976). Chem Rev.

[34] Li Z, Ji Y, Xie R, Grisham S Y, Peng X (2011). J Am Chem Soc.

[35] Cronyn M W, Jiu J (1952). J Am Chem Soc.

[36] Roy P K, Surekha P, Raman R, Rajagopal C (2009). Polym Degrad Stab.

[37] Pan D, Senpan A, Caruthers S D, Williams T A, Scott M J, Gaffney P J, Wickline S A, Lanza G M (2009). Chem Commun.

[38] Laurence C, Gal J-F (2010). Lewis Basicity and Affinity Scales: Data and Measurement.

[39] Mayr H, Ofial A R (2008). J Phys Org Chem.

[40] Mondini S, Cenedese S, Marinoni G, Molteni G, Santo N, Bianchi C L, Ponti A (2008). J Colloid Interface Sci.

[41] van Embden J, Chesman A S R, Jasieniak J J (2015). Chem Mater.

[42] Mourdikoudis S, Liz-Marzan L M (2013). Chem Mater.

[43] Baran J, Marchewka M K, Ratajczak H, Borovikov A Yu, Byckov V N, Naumovets A G, Podzelinsky A V, Puchkovskaya G A, Styopkin V I (1995). Thin Solid Films.

[44] Hironaka S, Yahagi Y, Sakurai T (1978). ASLE Trans.

[45] Klokkenburg M, Hilhorst J, Ernè B H (2007). Vib Spectrosc.

[46] Tjahjono M, Allian A D, Garland M (2006). Dalton Trans.

[47] Dluhy R A, Cornell D G (1985). J Phys Chem.

[48] Thomson J W, Nagashima K, Macdonald P M, Ozin G A (2011). J Am Chem Soc.

[49] Hyeon T, Lee S S, Park J, Chung Y, Na H B (2001). J Am Chem Soc.

