# Supplementary material for: Surfactant-controlled composition and crystal structure of manganese(II) sulfide nanocrystals prepared by solvothermal synthesis
Source: Beilstein J Nanotechnol. 2015 Dec 7;6:2319–29. doi: 10.3762/bjnano.6.238 (PMC4685872; doi:10.3762/bjnano.6.238)
Supplement: File 1 — Additional TEM images and ED patterns of MnO and MnS nanocrystals. [file Beilstein_J_Nanotechnol-06-2319-s001.pdf]

Supporting Information
for

Surfactant-controlled composition and crystal structure of manganese(II) sulfide nanocrystals prepared by solvothermal synthesis

Elena Capetti[1], Anna M. Ferretti[1], Vladimiro Dal Santo[2], and Alessandro Ponti[1,*§]

Address: [1]Laboratorio di Nanotecnologie, Istituto di Scienze e Tecnologie Molecolari, Consiglio Nazionale delle Ricerche, via G. Fantoli 16/15, 20138 Milano, Italy, and [2]Istituto di Scienze e Tecnologie Molecolari, Consiglio Nazionale delle Ricerche, via C. Golgi 19, 20133 Milano, Italy

Email: Alessandro Ponti - alessandro.ponti@istm.cnr.it

* Corresponding author

[§]Tel.: +39 02 5031 4280, Fax: +39 02 5031 3927

Additional TEM images and ED patterns of MnO and MnS nanocrystals

Morphology of MnO and MnS Nanocrystals

Being an in-depth discussion of NC morphology outside the scope of this paper, we limit ourselves to a brief summary of the size and shape of the MnO and MnS NC obtained using stearic acid (StAC) as a precursor. Morphological data are collected in Table S1 (TEM images of selected samples can be found in Figure 1 of the main text). MnO NCs prepared from $Mn_2(CO)_{10}$ have octahedral shape and size in the 10–20 nm range with dispersity 15–22%. Manganese monooleate gave somewhat larger and more disperse (15–27%) MnO NCs with shape similar to the MnO case.

MnO NCs usually have spherical or octahedral shape. Size and dispersity depend on the precursor. MnO NCs prepared from $Mn_2(CO)_{10}$ have size in the 10–20 nm range with dispersity 15–22%. Manganese monooleate gave somewhat larger and more disperse (15–27%) MnO NCs. Size and dispersity further increased when manganese dioleate ($MnOl_2$) was used and even further when the precursor was manganese distearate ($MnSt_2$).

The size of α-MnS NCs was in the 10–65 nm range, with dispersity 15–35%, as already observed [A. Puglisi; S. Mondini; S. Cenedese; A. M. Ferretti; N. Santo; A. Ponti, *Chem. Mater.* **2010,** 22, 2804-2813]. In most cases, α-MnS NCs display spherical or octahedral shape.

In general, considering that no effort was spent to optimize the synthetic conditions, the NCs size dispersion is acceptable and a remarkable shape uniformity was achieved.

Finally, it is noteworthy that manganese dicarboxylate precursors yielded multipodal MnO NCs when S/Mn < 1:1 was used. $MnOl_2$ resulted in multipodal NCs comprising up to 6 oval lobes (form factor = 1.6). When $MnSt_2$ was used, multipodal NCs (rods, T's, crosses) had more elongated branches (form factor = 3.6) with constant width and jagged edges. The analysis of MnO multipodes will be deferred to future publications.

Table S1: Properties of NCs synthesized by the thermal decomposition of a manganese precursor in octadecene containing varying amounts of sulfur (S) and stearic acid (L).

Precursor	S/Mn	L/Mn[a]	NC type	Shape[b]	Median diameter (nm)[c]	Diameter std. dev. (nm)[c]
$Mn_2(CO)_{10}$						
	1:1	1:1	MnO / α-MnS	octahedron	12	1.7
		2:1	MnO	octahedron	12	1.4
		3:1	MnO	octahedron	17	3.1
	2:1	2:1	MnO / α-MnS	octahedron	10	1.8
		3:1	MnO	octahedron	12	1.8
		4:1	α-MnS	octahedron	23	3.8
				sphere	8	1.2
	4:1	2:1	α-MnS	sphere	40	8.9
		3:1	α-MnS	sphere	28	3.3
Mn(OH)Ol [d]						
	0:5	1:1	MnO	IRC	63 × 10	31.4 × 5.8
		4:1	MnO	octahedron	25	6.7
	1.7 :1	0.6:1	α-MnS	sphere	14	2.3
	2:1	0:1	α-MnS	sphere	17.5	2.3
	2.3 :1	0.6:1	α-MnS	sphere	14.7	2.7
		1:1	MnO / α-MnS	sphere	20	4.9
				octahedron	25	6
	3:1	0:1	α-MnS	sphere	18	2.4
	4:1	0:1	α-MnS	sphere	16	2.8
$MnOl_2$						
	0:1	3:1	MnO	spheroidal	6	1.8
		4:1	MnO	octahedron	72	19
	0.5:1	0:1	MnO	IRC	45	12.2
		1:1	MnO	octahedron	46	7.2
		2:1	MnO	quasi-sphere	20	2.8
		3:1	MnO	4-flower	54	12.8
				octahedron	53	12.3
				T-shape	53 × 47	12.3 × 12.2
		4:1	MnO	crosses	80	16.4
				T-shape	82 × 56	16.1 × 16.1
				6-flower	77	16
		6:1	MnO	sphere	23	18
				T-shape	71 × 43	22.5 ×22.3
				flower-like	71	19.1
		7:1	MnO	quasi-sphere	34 × 21	10.6 ×10.5
		8:1	MnO	sphere	12	2.8
	2:1	0:1	α-MnS	sphere	19	2.5
		1:1	MnO/α-MnS	sphere	24	2.0
	3:1	0:1	α-MnS	sphere	21.6	2.9

		1:1	MnO/α-MnS	sphere	7	0.7
				sphere	22	6.9
				octahedron	21.5	4.3
	4:1	0:1	α-MnS	sphere	21.5	3.6
		1:1	α-MnS	sphere	21.9	3.0
MnSt2						
	0:5	0:1	MnO	IRC	55	9.2
		0:8	MnO	crosses	98	28.4
				T-shape	105 × 60	27.4 × 27.2
				rod	114	28.2
		1:1	MnO	crosses	80	24.3
				T-shape	79 × 45	24.5 × 24.4
				rod	73	21.7
		2:1	MnO	quasi-sphere	39	24.0
				T-shape	81 × 63	24.8 × 24.7
				rod	81	25.2
		3:1	MnO	IRC	25	5.9
		4:1	MnO	crosses	78	24
				T-shape	75 × 75	23.6 × 23.6
				rod	73	23.6
	2:1	0:1	α-MnS	quasi-sphere	65	9.9
				IRC	12 × 8	4 × 2
		1:1	α-MnS	quasi-sphere	7	1.2
		4:1	MnO/α-MnS	ellipse	31 × 23	4 × 3
	4:1	0:1	α-MnS	octahedron	29	8.7

[a]For $Mn_2(CO)_{10}$ and $MnSt_2$, L = stearic acid; for Mn(OH)Ol and $MnOl_2$, L = oleic acid.

[b]IRC = irregular, rounded, convex shape; quasi-sphere = shape very close to spherical.

[c]Both maximum and minimum values are shown for anisotropic shapes.

[d]Data are in part taken from A. Puglisi; S. Mondini; S. Cenedese; A. M. Ferretti; N. Santo; A. Ponti, *Chem. Mater.* **2010,** 22, 2804-2813.

TEM images and ED patterns of NCs prepared by thermal decomposition of manganese(II) distearate ($MnSt_2$) in the presence of sulfur (S) and different surfactants (L) with S/Mn = 2 and L/Mn = 4. See Table 1 in the main text.

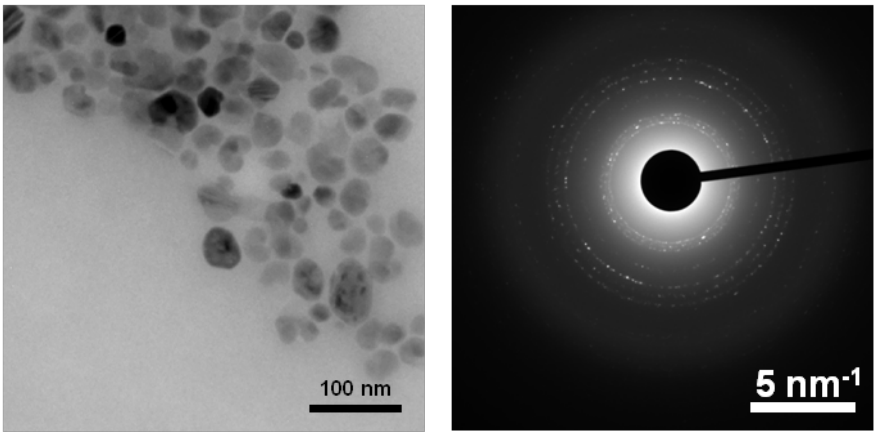

L = DdAm; outcome: γ-MnS NCs.

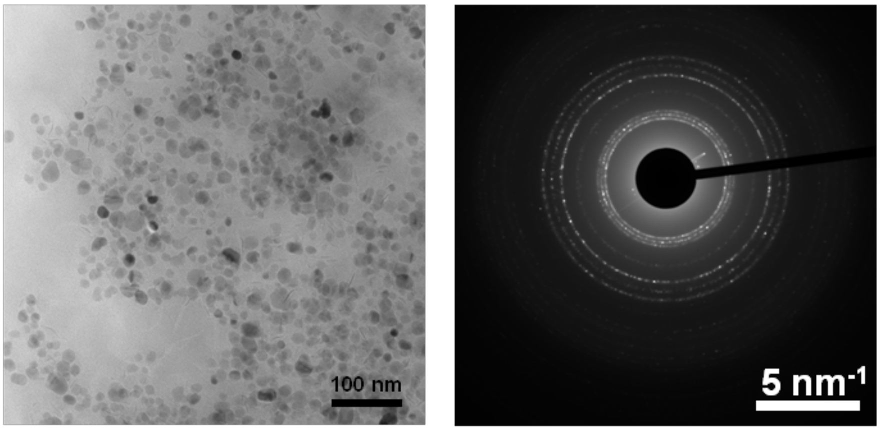

L = HdAm; outcome: γ-MnS NCs.

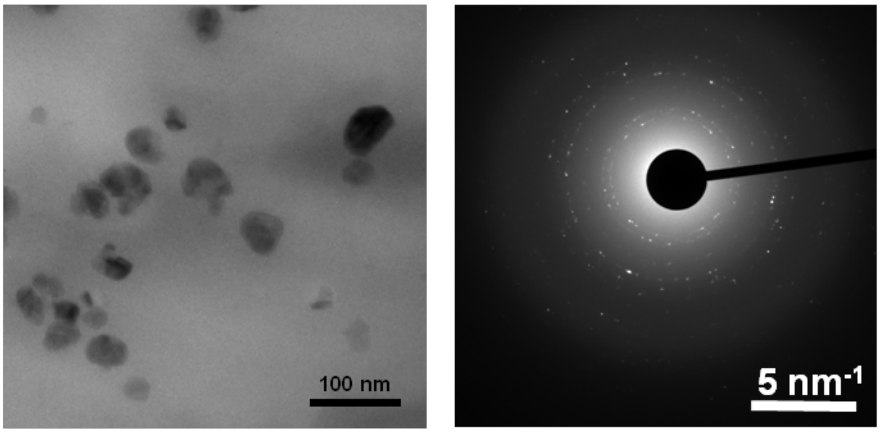

L = OdAm; outcome: γ-MnS NCs.

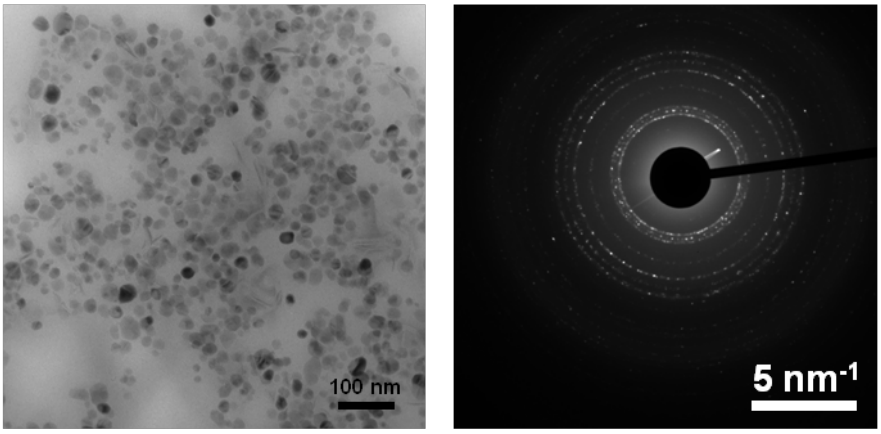

L = OlAm; outcome: γ-MnS NCs.

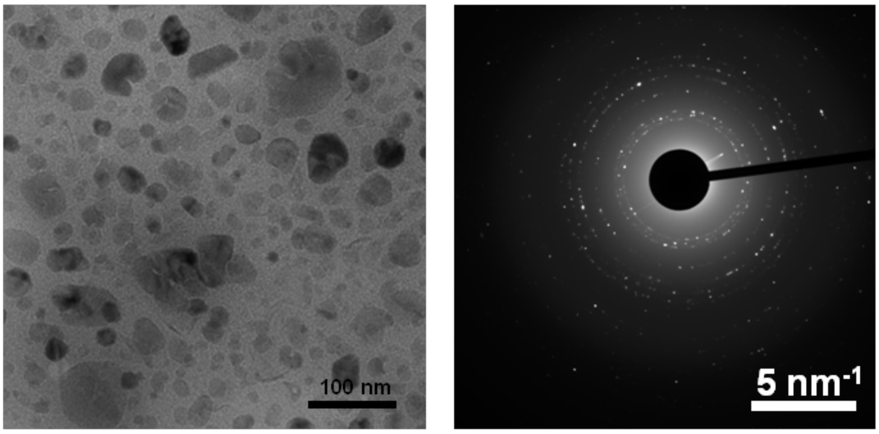

L = OlAm+ DdTh; outcome: γ-MnS NCs.

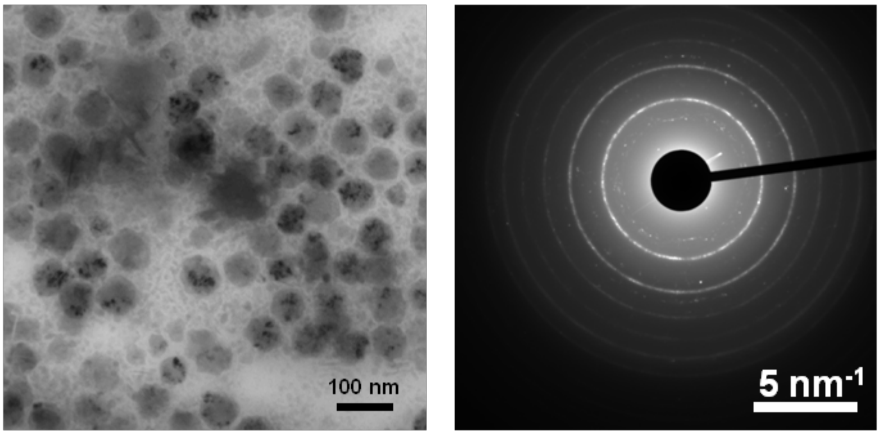

L = none; outcome: α-MnS NCs.

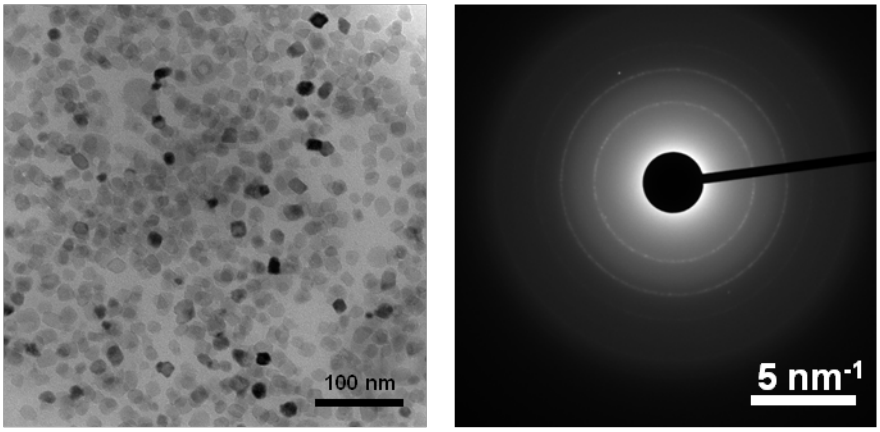

L = OlAlc; outcome: α-MnS NCs.

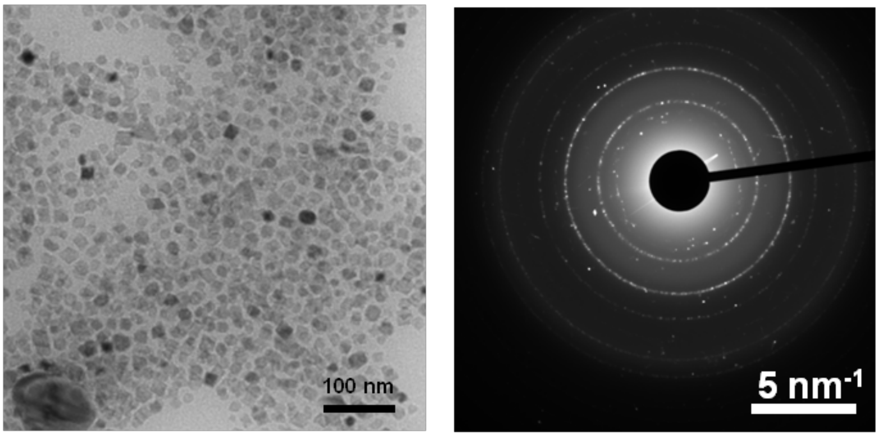

L = DdTh; outcome: α-MnS NCs.

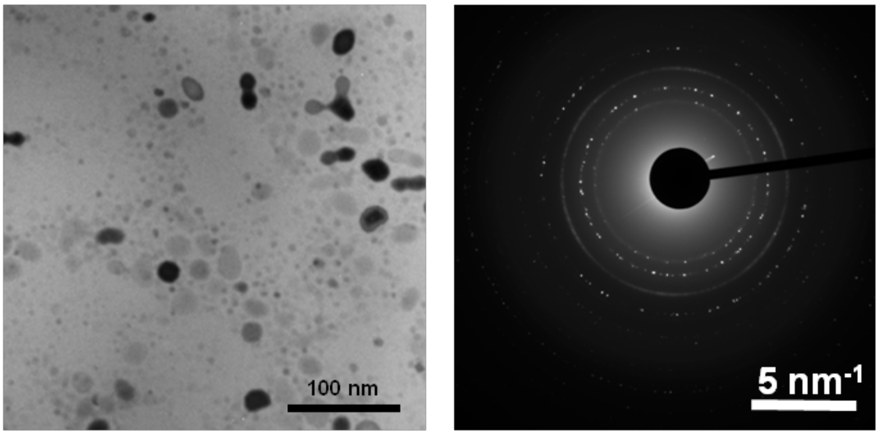

L = StAc; outcome: α-MnS NCs.

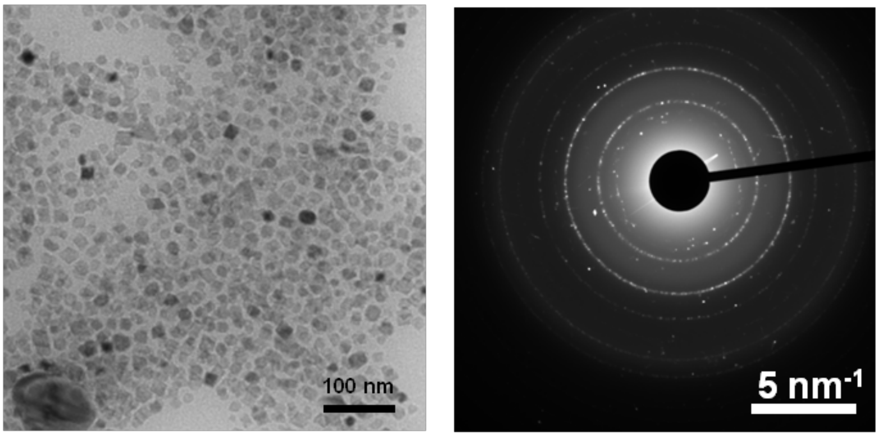

L = DdTh; outcome: α-MnS NCs.

TEM images and ED patterns of NCs prepared by thermal decomposition of manganese decacarbonyl [$Mn_2(CO)_{10}$] in the presence of sulfur (S) and different amine surfactants (L) with S/Mn = 2 and L/Mn = 4. See. Table 2 in the main text.

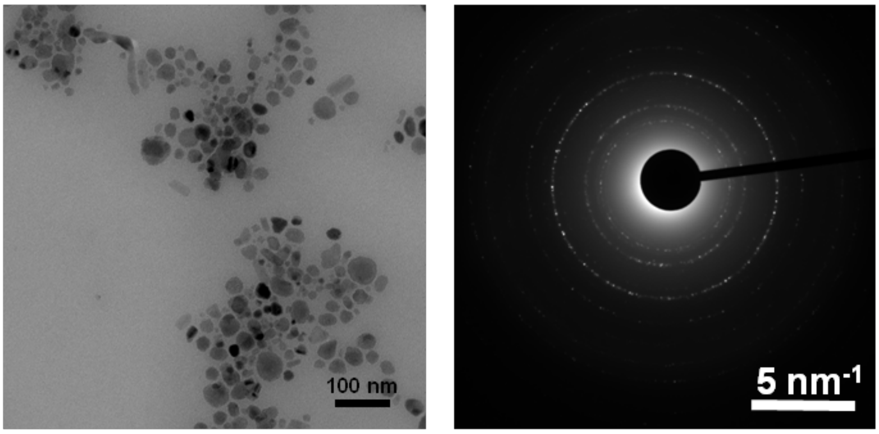

L = OlAm; outcome: α-MnS NCs.

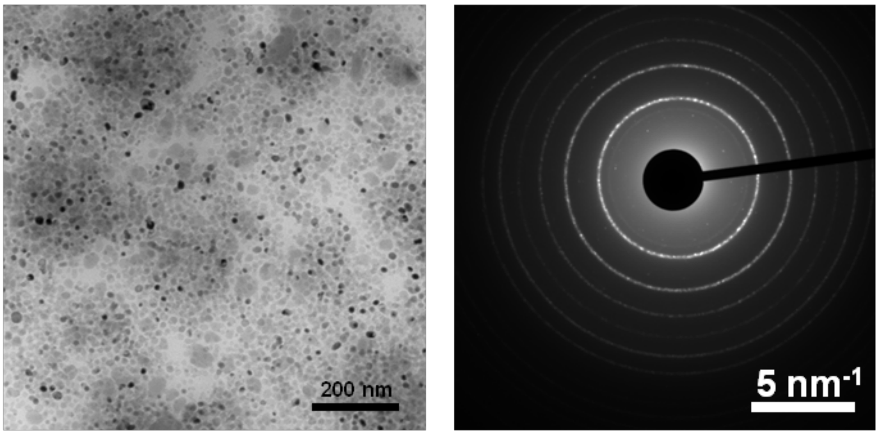

L = DdAm; outcome: α-MnS NCs.

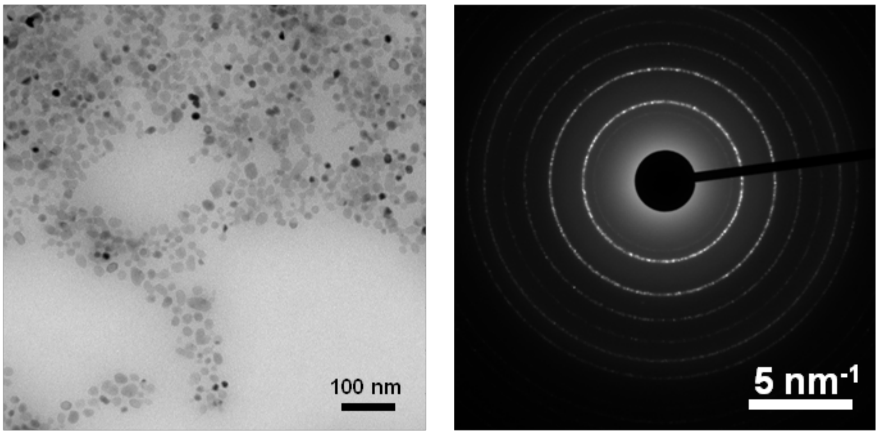

L = HdAm; outcome: α-MnS NCs.

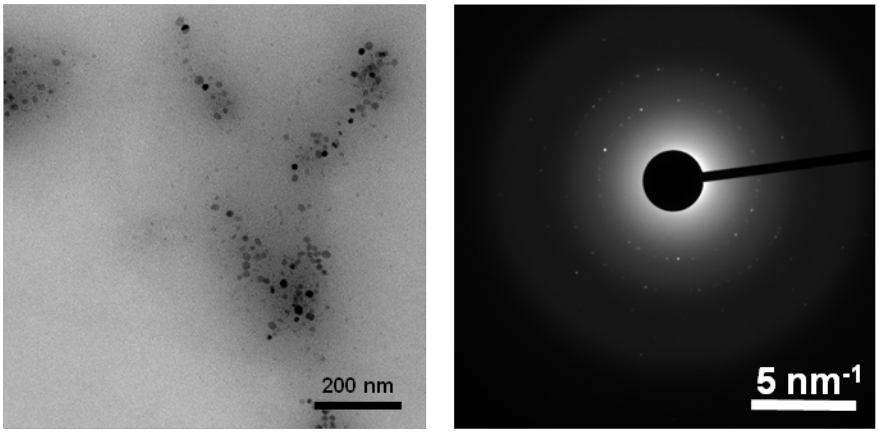

L = OdAm; outcome: α-MnS NCs.

TEM images and ED patterns of NCs prepared by thermal decomposition of manganese decacarbonyl [$Mn_2(CO)_{10}$] in the presence of sulfur (S) and a mixture of carboxylic acid (L_{acid}) and amine (L_{amine}) surfactants with S/Mn = 2, L_{acid}/Mn = 2 and L_{amine}/Mn = 4. See Table 3 in the main text.

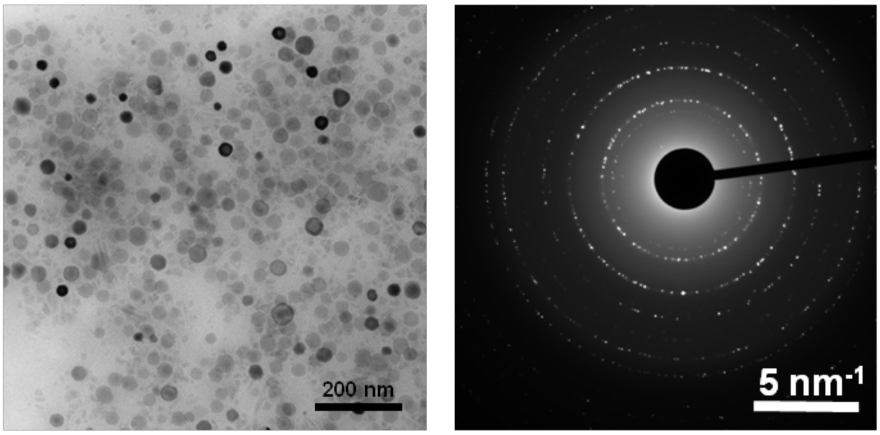

L_{amine} = OlAm, L_{acid} = StAc; outcome: γ-MnS NCs.

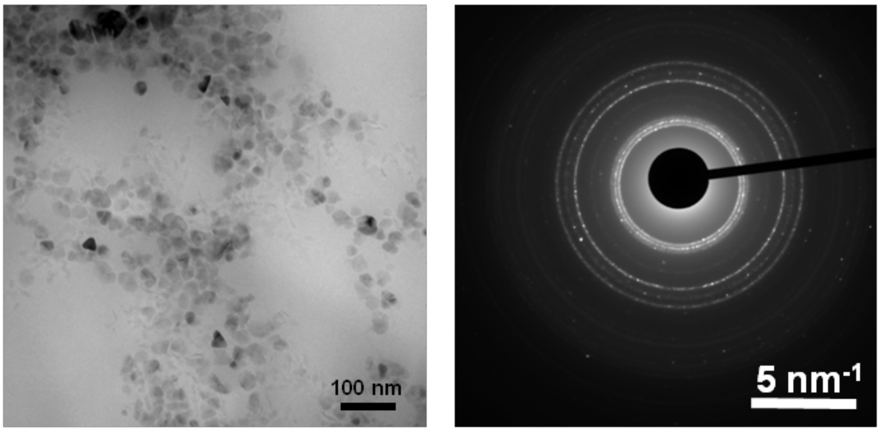

L_{amine} = HdAm, L_{acid} = StAc; outcome: γ-MnS NCs.

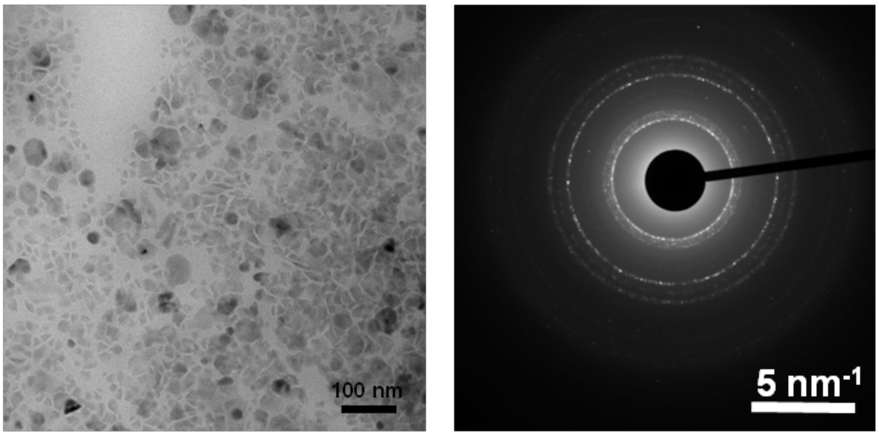

L_{amine} = DdAm, L_{acid} = StAc; outcome: γ-MnS NCs.

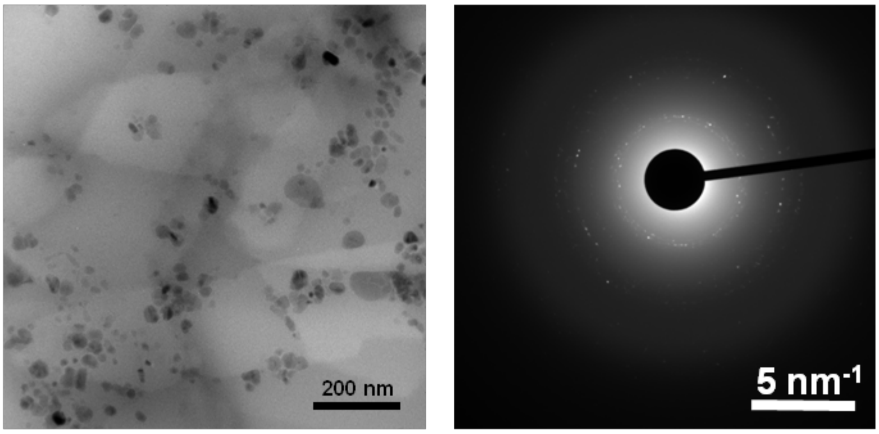

L_{amine} = OdAm, L_{acid} = StAc; outcome: γ-MnS NCs.

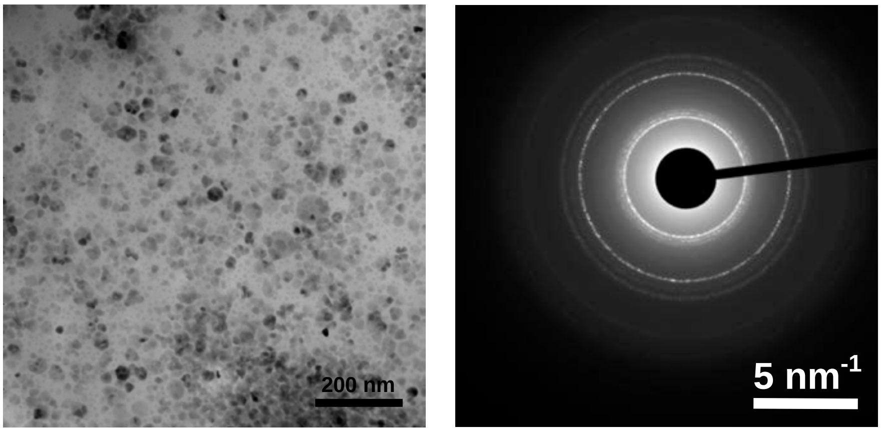

L_{amine} = HdAm, L_{acid} = OlAc; outcome: γ-MnS NCs.
